# House mouse *Mus musculus* dispersal in East Eurasia inferred from 98 newly determined complete mitochondrial genome sequences

**DOI:** 10.1038/s41437-020-00364-y

**Published:** 2020-09-15

**Authors:** Yue Li, Kazumichi Fujiwara, Naoki Osada, Yosuke Kawai, Toyoyuki Takada, Alexey P. Kryukov, Kuniya Abe, Hiromichi Yonekawa, Toshihiko Shiroishi, Kazuo Moriwaki, Naruya Saitou, Hitoshi Suzuki

**Affiliations:** 1 Graduate School of Environmental Science, Hokkaido University, North 10, West 5, Kita-ku, Sapporo, 060-0810 Japan; 2 Graduate School of Information Science and Technology, Hokkaido University, North 14, West 9, Kita-ku, Sapporo, 060-0814 Japan; 3 Global Station for Big Data and Cybersecurity, GI-CoRE, Hokkaido University, North 14, West 9, Kita-ku, Sapporo, 060-0814 Japan; 4 Genome Medical Science Project (Toyama), National Center for Global Health and Medicine, 1-21-1, Toyama, Shinjuku-ku, Tokyo, 162-8655 Japan; 5 Integrated Bioresource Information Division, RIKEN BioResource Research Center, 3-1-1 Koyadai, Tsukuba, 305-0074 Japan; 6 Far Eastern Branch of the Russian Academy of Sciences, Federal Scientific Center of the East Asia Terrestrial Biodiversity, Vladivostok, 690022 Russia; 7 Technology and Development Team for Mammalian Genome Dynamics, RIKEN BioResource Research Center, 3-1-1 Koyadai, Tsukuba, 305-0074 Japan; 8 Laboratory for Transgenic Technology, Tokyo Metropolitan Institute of Medical Science, 2-1-6 Kami-kitazawa, Setagaya-ku, Tokyo, 156-8506 Japan; 9 RIKEN BioResource Research Center, 3-1-1 Koyadai, Tsukuba, 305-0074 Japan; 10 National Institute of Genetics, 1111 Yata, Mishima, 411-8540 Japan; 11 Population Genetics Laboratory, National Institute of Genetics, 1111 Yata, Mishima, 411-8540 Japan; 12 School of Medicine, University of the Ryukyus, 207 Uehara, Nishihara-cho, 903-0215 Japan

**Keywords:** Evolution, Genetic variation

## Abstract

The Eurasian house mouse *Mus musculus* is useful for tracing prehistorical human movement related to the spread of farming. We determined whole mitochondrial DNA (mtDNA) sequences (ca. 16,000 bp) of 98 wild-derived individuals of two subspecies, *M*. *m*. *musculus* (MUS) and *M*. *m*. *castaneus* (CAS). We revealed directional dispersals reaching as far as the Japanese Archipelago from their homelands. Our phylogenetic analysis indicated that the eastward movement of MUS was characterised by five step-wise regional extension events: (1) broad spatial expansion into eastern Europe and the western part of western China, (2) dispersal to the eastern part of western China, (3) dispersal to northern China, (4) dispersal to the Korean Peninsula and (5) colonisation and expansion in the Japanese Archipelago. These events were estimated to have occurred during the last 2000–18,000 years. The dispersal of CAS was characterised by three events: initial divergences (ca. 7000–9000 years ago) of haplogroups in northernmost China and the eastern coast of India, followed by two population expansion events that likely originated from the Yangtze River basin to broad areas of South and Southeast Asia, including Sri Lanka, Bangladesh and Indonesia (ca. 4000–6000 years ago) and to Yunnan, southern China and the Japanese Archipelago (ca. 2000–3500). This study provides a solid framework for the spatiotemporal movement of the human-associated organisms in Holocene Eastern Eurasia using whole mtDNA sequences, reliable evolutionary rates and accurate branching patterns. The information obtained here contributes to the analysis of a variety of animals and plants associated with prehistoric human migration.

## Introduction

The early to mid-Holocene is a crucial period in the development of present day human cultural and genetic diversity. The house mouse (*Mus musculus*) is a ubiquitous human commensal that is known to have spread through almost all of Eurasia with prehistoric human movements. The temporal and spatial aspects of the dispersal of mice, therefore, provide a practical way to interpret the process of human migration, agricultural development and cultural exchanges (Sage 1981; Moriwaki et al. 1986; Bonhomme et al. 2011; Gabriel et al. 2011; Jones et al. 2013). However, the dispersal events of *M*. *musculus* during the Neolithic period are complex, and archaeological data remain poorly understood, partly because archaeological excavations of small mammals, such as house mice, are often neglected.

The evolutionary history of *M*. *musculus* has been predicted from a variety of molecular phylogenetic markers, including nuclear and mitochondrial gene sequences (e.g., Moriwaki et al. 1986; Yonekawa et al. 1988; Bonhomme et al. 1989; Boursot et al. 1993; Rajabi-Maham et al. 2008, 2012; Kodama et al. 2015). Based on previous molecular phylogenetic studies, *M*. *musculus* is composed of three major subspecies groups: *M*. *m*. *castaneus* (CAS), *M*. *m*. *domesticus* (DOM) and *M*. *m*. *musculus* (MUS). These groups are believed to have originated in southern and western Asia, where they had a parapatric distribution in the central, western and northern portions of their home range, respectively (Britton and Thaler 1978; Sage 1981; Bonhomme et al. 1984; Boursot et al. 1993; Prager et al. 1998; Kodama et al. 2015). The three subspecies are known to have settled in their present geographic territories due to human activities from the Neolithic onwards, with DOM ranging from the Middle East to western Europe, CAS from Iran to southern China and Indonesia and MUS from the southern coastal region near the Caspian Sea through eastern Europe and northern Eurasia, including western and northern China, the Korean Peninsula and the Japanese Archipelago (e.g., Duplantier et al. 2002; Bonhomme et al. 2007; Suzuki et al. 2013). DOM currently has a wide distribution covering Africa, Oceania and North and South America and can be easily introduced anywhere in the world (e.g., Gabriel et al. 2011). Five distinct lineages of mitochondrial DNA (mtDNA) exist in *M*. *musculus* (Prager et al. 1998; Sakuma et al. 2016), three of which represent the three major subspecies groups of MUS, DOM and CAS and two of haplotypes from limited geographic areas in Nepal (Terashima et al. 2006) and Yemen and Madagascar (Duplantier et al. 2002), respectively. The three major subspecies lineages contain substantial levels of divergence within each of them.

The mtDNA polymorphism of wild mice has been used for tracking their movements (Yonekawa et al. 1988; Bonhomme et al. 1989; Boursot et al. 1993; Rajabi-Maham et al. 2008, 2012; Suzuki et al. 2013; Jing et al. 2014) and assessing their population dynamics (Gündüz et al. 2005; Suzuki et al. 2013; Kuwayama et al. 2017). Based on a number of previous mtDNA studies, MUS and CAS are recognised as the representative subspecies of northern Eurasia and South Asia, respectively, and the majority of the prehistoric movements of each have been carried out by a single sublineage (termed MUS-1 and CAS-1; Suzuki et al. 2013). MUS-1 is predicted to have migrated from the Korean Peninsula to the Japanese Archipelago ca. 2000 years ago (Kuwayama et al. 2017), while CAS-1 has been suggested to have travelled from southern China to surrounding areas, including the Japanese Archipelago, ca. 4000 years ago (Suzuki et al. 2013; Kuwayama et al. 2017). Both mtDNA and nuclear DNA markers indicate that MUS and CAS comprise major and minor components of the mouse lineages present in Japan, respectively (Minezawa et al. 1979; Bonhomme et al. 1984; Yonekawa et al. 1988; Terashima et al. 2006; Nunome et al. 2010; Kodama et al. 2015). The detailed routes and dates of the eastward migration of MUS-1 and CAS-1 have not yet been precisely determined. Moreover, the historical movements of *M*. *musculus* in Asia have not been fully clarified using multiple lines of archaeological evidence (e.g., Crawford and Lee 2003; Fuller et al. 2010, 2014; Fuller 2011).

The mtDNA marker, which has played a significant role in tracing the phylogeographic history and population dynamics of *M*. *musculus*, as mentioned above, is useful because of its rapid evolution and lack of recombination, allowing genealogical patterns to be traced and divergence times to be estimated. Short fragments of mtDNA markers, such as the regulatory region (*D-loop*), cytochrome *b* (*Cytb*) and NADH dehydrogenase subunit 3 (*Nd3*) genes, have been used in previous studies (Nachman et al. 1994; Prager et al. 1998; Suzuki et al. 2013; Jing et al. 2014; Bibi et al. 2017). However, their spatiotemporal dynamics remain poorly understood, presumably due to the small sizes of the markers, leading to insufficient resolution for identifying intricate branching patterns. To overcome these problems, complete mtDNA genome sequences (hereafter mitogenomes) could provide useful phylogeographic markers, as exemplified in studies of humans (*Homo sapiens*; Ingman et al. 2000; Sahakyan et al. 2017), brown rats (*Rattus norvegicus*; Puckett et al. 2018) and killer whales (*Orcinus orca*; Morin et al. 2010).

Another problem in previous studies using mtDNA markers is that their evolutionary rate, which is essential for accurate temporal assessment of lineage differentiation, remains unclear. Fossil evidence from late Pleistocene deposits is limited. Moreover, mtDNA evolutionary rates are time dependent over short time scales (Ho and Larson 2006; Ho et al. 2011), indicating the need for multiple calibration points to assess the differing evolutionary rates over time. Meanwhile, recent studies using multiple biogeographic calibration points have identified the time-dependent evolutionary rates of house mice (Rajabi-Maham et al. 2008; Förster et al. 2009), wood mice (Suzuki et al. 2015; Hanazaki et al. 2017), voles (Honda et al. 2019) and flying squirrels (Oshida et al. 2009). In natural populations of rodents, over the last 150,000 years, three episodic periods have occurred that could have fostered rapid expansion events, in which bottleneck events during extensive glaciation periods and the subsequent population expansions during rapid warming periods are linked, namely the transitions from marine isotope stage (MIS) 6 to MIS 5e (ca. 135,000 years ago), from MIS 4 to early MIS 3 (ca. 53,000 years ago) and from MIS 2 to MIS 1 (ca. 11,000 years ago) (Hanazaki et al. 2017; Honda et al. 2019).

In this study, we determined the entire mitogenome sequences of 98 individuals collected across a broad geographic area covering 16 countries in Eurasia, which included four major mtDNA lineages of *M*. *musculus*: MUS, CAS, DOM and that from Nepal (NEP). We focused on the evolutionary history of the eastward movements of MUS-1 and CAS-1 in particular and aimed to precisely infer the divergence patterns and times of the major mtDNA lineages and elucidate the detailed spatiotemporal dynamics of MUS and CAS in northern and southern Eurasia.

## Materials and methods

### Materials

**Fig. 1:**
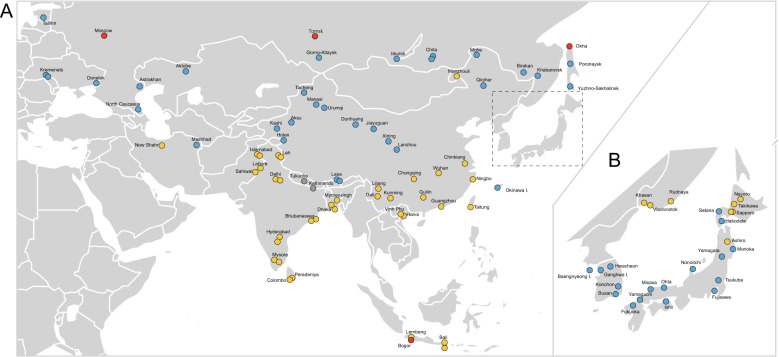
Map showing the collection locations of samples used in this study. The range extends across Eurasia (**a**) and the Japanese Archipelago (**b**). The colours of circles indicate subspecies based on the mitochondrial genotyping performed in this study: *Mus musculus musculus* (MUS, blue), *M*. *m*. *castaneus* (CAS, yellow), *M*. *m*. *domesticus* (DOM, red) and a geographically confined group of Nepalese mice (NEP, grey).

We used a total of 98 house mouse samples in this study. Most of our samples overlap with those used by Suzuki et al. (2013). The samples were collected throughout Eurasia, representing 16 regions, as shown in Fig. 1 and listed in Supplementary Table S1.

### DNA extraction and variant calling

We determined the whole mitogenome sequences of the 98 house mouse samples (ca. 16,000 bp). Our samples, along with their qualified concentrations and volumes, were sent to BGI (Shenzhen, China) for whole-genome sequencing. Libraries were constructed for each sample with index sequences, and paired-end reads of 100 bp were sequenced using the BGISEQ-500 platform by BGI. For each sample, ~1 billion clean reads were obtained. We mapped the raw reads to the GRCm38 (mm10) house mouse reference genome sequence, including the mitogenome, using the BWA-MEM method (Li and Durbin 2009) with the ‘-M’ command option. Samblaster (Faust and Hall 2014) (https://github.com/GregoryFaust/samblaster) with the ‘-M’ command option was used for identifying duplicates in read-id groups for exclusion from downstream analysis. The average median coverage of the whole genome sequence was 30.4 per sample. When reads were simultaneously mapped to the nuclear genome and mitogenome, the reads mapped to regions of the mitogenome that were highly similar or identical to regions in the nuclear genome, due to nuclear mtDNA segments, and yielded very low mapping quality (MQ) scores. To recalibrate the MQ score, we remapped all mapped mitogenome reads to the C57BL/6J complete mitogenome (NC_005089.1) using BWA-MEM and recalculated the MQ scores. Single-nucleotide polymorphisms and indels were obtained using the GATK4 HaplotypeCaller program (Mckenna et al. 2010) following the ‘Best Practice’ pipeline instructions. Each gVCF file was merged using GenotypeGVCFs to simultaneously call the genotypes of all samples. To identify low-depth uncalled sites, we created a consensus sequence in FASTA format using bcftools consensus with the ‘-M’ option to determine missing genotypes.

### Sequence analysis

For further analysis, the original sequences were aligned using MUSCLE implemented in MEGA7 (Kumar et al. 2016) to ensure the consistency of the mitogenome sequences and to remove alignment gaps (16,038 bp). We downloaded the mitogenome reference sequence of *Mus spretus* from a public database (accession number: NC_025952, Chang et al. 2016) and obtained aligned mitogenome sequences (15,185 bp).

### Phylogeny and divergence time estimation

A network of the complete mitogenome sequences was constructed using the neighbour-net (NN) method (Bryant and Moulton 2004), as implemented in SplitsTree4 software (v4.14.8) (Huson and Bryant 2006), and the mitogenome dataset (*n* = 98, 16,038 bp). The principal advantage of this hypothesis-poor method over others that generate dichotomous branching networks or trees is that NN networks illustrate all potentially supported splits among a group of sequences as a reticulation. Further insights into the structure of each subgroup was obtained by constructing networks with mitogenome sequences (16,038 bp) using the median-joining (MJ) method (Bandelt et al. 1999), as implemented in PopART software (Leigh and Bryant 2015). Maximum likelihood (ML) trees were constructed using MEGA7 software (Kumar et al. 2016) based on the mitogenome sequences (*n* = 99, 15,185 bp), MUS (*n* = 47) and CAS (*n* = 44) datasets, with substitution modes of GTR + G + I, HKY + G and HKY + G + I, respectively, which were determined using the Akaike information criterion, as implemented in MEGA. We performed 1000 bootstrap replicates to assess the robustness at each node. The time to the most recent common ancestor (tMRCA) of the mitogenome sequences (16,038 bp) and 95% highest posterior density (HPD) was estimated using BEAST v1.8.4 software (Drummond and Rambaut 2007). The Markov chain Monte Carlo simulation was run for 10,000,000 generations and sampled every 10,000 generations. The first 1000,000 generations were discarded as the burn-in period. Tracer v1.6 software (Rambaut and Drummond 2009) was used to assess the convergence of Markov chain Monte Carlo chains. All parameters had effective sample sizes >200. The trees were summarised using TreeAnnotator v1.8.4 software (http://beast.community/treeannotator) with the settings “Maximum clade credibility tree” and “Mean heights”, and were displayed using FigTree v1.4.3 software (http://tree.bio.ed.ac.uk/software/figtree/).

The time-dependent evolutionary rates of mtDNA (Ho et al. 2011) were taken into account in this study. Various evolutionary rates of *Cytb* have been reported: 3.0 × 10^−8^ (lower rate) and 1.1 × 10^−7^ substitutions/site/year (higher rate) for ancient (>100,000 years ago) and recent (i.e., <20,000 years ago) divergences, respectively (Suzuki et al. 2015; Hanazaki et al. 2017; Honda et al. 2019). In addition, an intermediate evolutionary rate of 4.7 × 10^−8^ substitutions/site/year has been proposed (Hanazaki et al. 2017) for an intermediate time period (50,000–60,000 years ago). To assess the standard evolutionary rate of the mitogenome for older divergences, the genetic distances of mitogenome and *Cytb* sequences were determined and compared using our current mitogenome data from 98 samples. The former was roughly 80% of that from the latter (3.0 × 10^−8^), suggesting an evolutionary rate of 2.4 × 10^−^^8^ substitutions/site/year for the mitogenome (Supplementary Fig. S1). For younger divergences (i.e., <20,000 years ago), we used 1.1 × 10^−^^7^ substitutions/site/year, together with 0.9 × 10^−7^ substitutions/site/year (80% rate of 1.1 × 10^−^^7^), as possible evolutionary rates. Furthermore, we considered alternative higher rates of 2.0 × 10^−^^7^ and 4.0 × 10^−^^7^ substitutions/site/year, as high rates are often used in studies on the population dynamics of *M. musculus* (e.g., Rajabi-Maham et al. 2008; García-Rodríguez et al. 2018).

### Assessment of historical demographical processes

The number of haplotypes (*H*), haplotype diversity (Hd), number of polymorphic sites (*S*), nucleotide diversity (*π*) and mean number of pairwise differences among sequences (*K*) were estimated using DnaSP software (v5.00.7) (Librado and Rozas 2009). Tajima’s *D* and Fu’s *F*s values were estimated with ARLEQUIN 3.5.1 software (Excoffier and Lischer 2010). The pairwise mismatch distributions (Rogers and Harpending 1992), which comprise the pairwise differences among all individuals within each clade, were compared using the simulated sudden expansion model, and population demographic parameters were estimated using ARLEQUIN 3.5.1 software (Excoffier and Lischer 2010). The expected distribution was simulated for the sudden expansion model through mismatch distribution analysis, in which the validity of the model was tested using a parametric bootstrap approach with 1000 replicates. In this method, the sum of the squared deviations (SSD) between the observed distribution and the expected distribution was compared with the SSD between the simulated distribution and the expected distribution for each replicate. The raggedness index (*r*; Harpending 1994) was used as a test statistic for the predicted sudden expansion model. The temporal aspect of rapid expansion was assessed using the formula *t* = *τ*/2*uk*, where *t* is the time since expansion in generations, *τ* is a unit of mutational time, *k* is the sequence length and *u* is the evolutionary rate per generation for the entire sequence (Rogers and Harpending 1992; Rogers 1995). The value of *u* was derived from the formula *u* = *μg*, where *μ* is the evolutionary rate per site per year and *g* is the generation time in years. Time since expansion in years, *T* (=*tg*), was estimated using the formula *T* = *τ*/2*μk*.

## Results

### Phylogenetic analysis of major lineages within and between subspecies

We determined the entire mitogenome sequences of 98 individuals from Eurasia (Supplementary Table S1). A NN network constructed from the mitogenome sequences yielded four distinct lineages, designated CAS, DOM, MUS and NEP, representing the subspecies *M*. *m*. *castaneus*, *M*. *m*. *domesticus*, *M*. *m*. *musculus* and a hitherto unknown lineage (NEP) collected from Nepal, respectively (Supplementary Fig. S2). The basic genetic properties of the haplotypes in each subspecies group are summarised in Table 1. In the NN network, the CAS and MUS lineages showed three (CAS-1, CAS-2, CAS-3) and two (MUS-1, MUS-2) sublineages with bush-like terminal segments, respectively. While a single distinct haplotype was treated as ‘CAS-4’ previously (Suzuki et al. 2013), we integrated it into CAS-3 in this study. The four major lineages were equally divergent from a single central node, which we designated Node a. Similarly, the three CAS sublineages were equally divergent from a single node, which we designated Node b.

**Table 1:** Summary of populations, haplotypes and nucleotide diversity of *Mus musculus*.

Lineage group	*n*	*H*	*Hd*	*S*	*Pi* (%)	*K*
MUS	47	45	0.998	493	0.321	51.514
CAS	44	38	0.993	573	0.554	88.927
DOM	5	5	1	189	0.552	88.5
NEP	2	2	1	102	0.636	102

*H* number of haplotypes, *Hd* haplotype diversity, *S* number of segregating sites, *Pi* nucleotide diversity, *K* mean number of nucleotide differences.

**Fig. 2:**
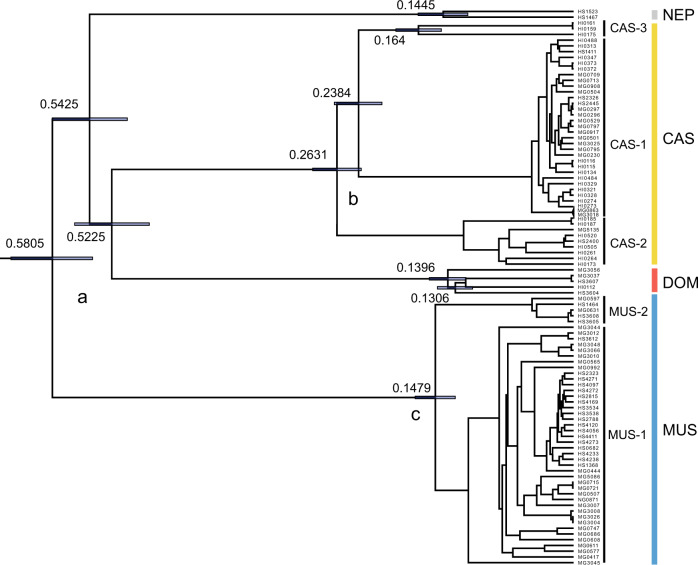
Divergence time estimates (million years ago [mya]) of *M*. *musculus* phylogroups, based on a Bayesian-relaxed molecular clock of 2.4 × 10^−^^8^ substitutions/site/year. The nodes corresponding to the most recent common ancestors of *M*. *musculus*, *M*. *m*. *castaneus* and *M*. *m*. *musculus* are marked with the letters a, b and c, respectively. Given the time-dependency of the mtDNA evolutionary rate, we considered only deeper nodes.

We performed BEAST analysis and estimated the divergence times of Nodes a and b, along with the divergence time between MUS-1 and MUS-2 (Node c). We obtained estimated divergence times of 520,000–580,000, 230,000–260,000 and 150,000 years ago for Nodes a, b and c, respectively (Fig. 2), using the lower evolutionary rate of 2.4 × 10^−8^ substitutions/site/year, which has been reported to cover divergence times prior to 130,000 years ago.

### Analysis of geographic structures of MUS and CAS

**Fig. 3:**
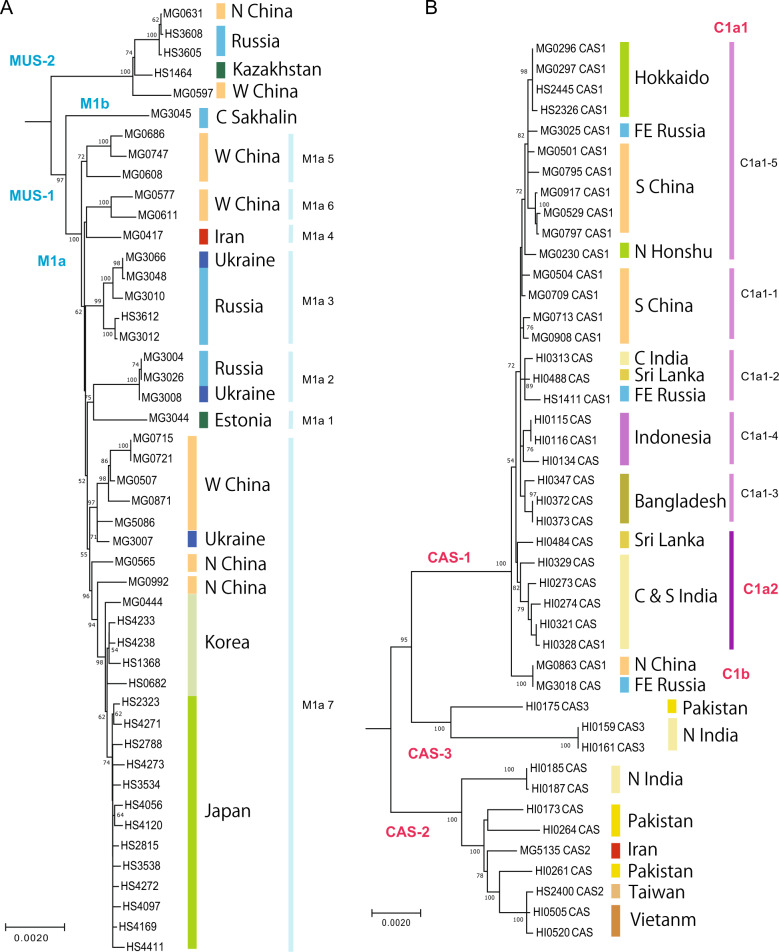
Maximum likelihood (ML) trees based on the whole mitochondrial sequences of *Mus musculus*. The phylogroups represnt the two subspecies groups of *M. m. musculus* (MUS) (**a**) and *M. m. castaneus* (CAS) (**b**). The sublineages MUS-1 and CAS-1, which were responsible for the eastward movements, are shown with their major haplogroups. Bootstrap support values >50% are indicated above the nodes. The colours of the vertical bars associated with the sample codes indicate the countries where the samples were collected.

The ML tree (Fig. 3) revealed further divisions of MUS-1 and CAS-1 into multiple monophyletic groups. The MUS-1 lineage recovered from Central Sakhalin (sample code: MG3045) had a distinct haplotype (M1b), with a high supporting value (100%) for the basal position. The tree revealed seven distinct lineages, termed M1a1–M1a7. The majority of CAS-1 haplotypes were grouped into the major cluster C1a, excluding distinct haplotypes from northern China and the Russian Far East (Clb). The haplotypes of C1a formed major (C1a1) and minor (C1a2) groups, with the latter represented by samples from central and southern India and Sri Lanka. The major haplogroup C1a1 included an internal cluster (C1a1-5) comprised of haplotypes from southern China (Yunnan etc.), Japan (Hokkaido and northern Honshu) and the Russian Far East.

**Fig. 4:**
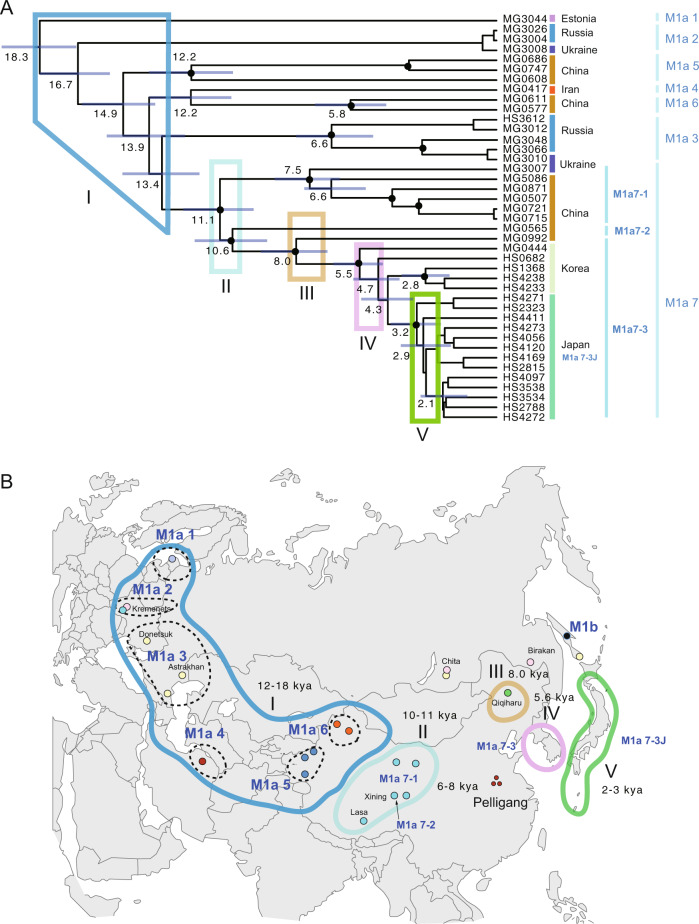
Assessment of genetic variation of the MUS-1 sublineage. **a** Divergence time estimates were determined using BEAST software. A strict clock of 1.1×10^−^^7^ substitutions/site/year was applied to the whole mitochondrial sequence (16,038 bp). The node ages and 95% highest posterior density intervals (blue vertical bars) of node ages within 1000 years are shown for nodes with particularly ancient divergence times. Nodes with >50% bootstrap values (Fig. 3a) are marked with black circles. The colours of the vertical bars associated with the sample codes indicate the countries where the samples were collected. The eastward movement of MUS-1 can be characterised by five steps (I–V): (1) broad spatial expansion into eastern Europe and western part of western China, (2) dispersal to the eastern part of western China, (3) dispersal to northern China, (4) dispersal to the Korean Peninsula and (5) colonisation and expansion in the Japanese Archipelago. The first two steps (I and II) may be correlated with the end of the last Ice Age (ca. 15,000 years ago) and the end of the Younger Dryas (11,600 years ago), respectively. **b** Geographic distribution of the seven earliest emerging haplogroups (M1a1–M1a7), as defined by the ML phylogenetic tree (Fig. 3). M1a7, the haplogroup located on the eastern edge of western China, experienced further range extension into northern China, Korea (M1a7-3) and Japan (M1a7-3J), sequentially. The times of the emergence of haplogroups were estimated in the BEAST analysis, except for M1a7-3J, which was obtained based on the mismatch distribution analysis (Table 2).

The tMRCAs of MUS-1 mitogenome sequences were estimated using BEAST software (Fig. 4a). The results revealed five notable times with distinctive spatial dynamics during the lineage differentiation of MUS-1: (I) the initial divergence among the seven major haplogroups M1a1–M1a7 with a range covering eastern Europe and western China, (II) divergence in the eastern part of western China, (III) lineage diversification in northern China, (IV) introduction of a lineage to the Korean Peninsula from northern China and (V) colonisation to the Japanese Archipelago from the Korean Peninsula and subsequent sudden expansion (Fig. 4b). The approximate times of the five historical events were calculated as 13,400–18,300, 10,600–11,100, 6600–8000, 4300–5500 and 2100–3200 years ago, respectively, using evolutionary rates of 1.1 × 10^−^^7^ substitutions/site/year, but the associated credibility intervals were notably high (horizontal blue lines in Fig. 4a). Three haplogroups of M1a5, M1a6 and M1a7 were recovered from western China and the adjacent area of northern China. Haplotypes from China (Qiqihar, MG0992), Korea and Japan (bootstrap value = 98%, M1a7-3) and those from Japan (bootstrap value = 74%, M1a7-3J) were found to form monophyletic groups (Fig. 3a).

**Fig. 5:**
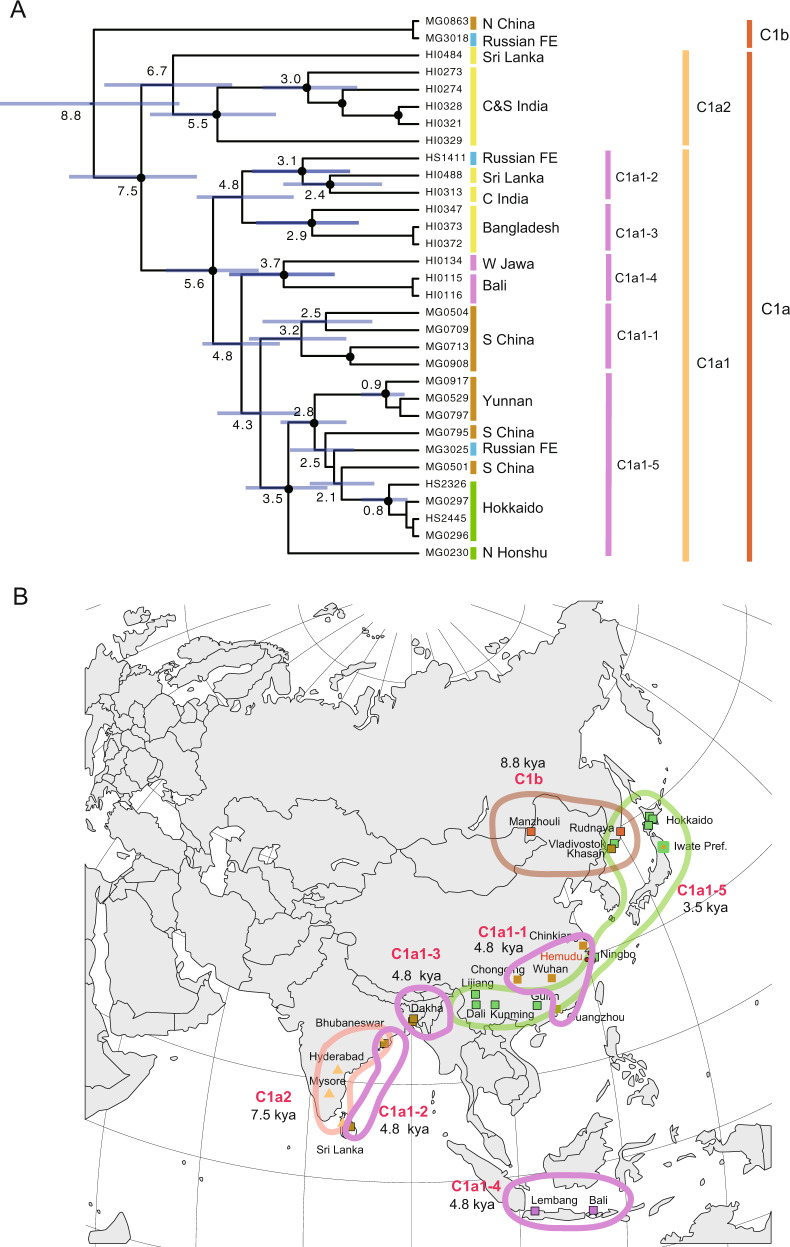
Assessment of genetic variation of the CAS-1 sublineage. **a** Divergence time estimates were determined using BEAST software. A strict clock of 1.1×10^−^^7^ substitutions/site/year was applied to the whole mitochondrial sequence (16,038 bp). The node ages and 95% highest posterior density intervals (blue vertical bars) of the node ages within 1000 years are shown for nodes with particularly ancient divergence times. Nodes with >50% bootstrap values (Fig. 3b) are marked with black circles. The colours of the vertical bars associated with the sample codes indicate the countries where the samples were collected. **b** Distribution of three haplogroups of CAS-1 revealed through phylogenetic analysis. The C1a1 haplotypes were recovered from a broad range of geographic areas across India, Indonesia and China. The next clade to have emerged, termed C1a1-5, extends from the Yangtze River basin in southern China to Japan.

BEAST analysis of the CAS-1 dataset indicated early divergence of the haplotype (C1b) from northernmost China and the Russian Far East with an estimated divergence time of 8800 (95% HPD: 6,800–11,500) years ago (Fig. 5). The remaining haplotypes formed the cluster C1a, which split into two clades, C1a1 and C1a2, the latter of which was recovered from the eastern coasts of India and Sri Lanka. C1a1 consists of five haplogroups, C1a1-1–C1a1-5, covering a broad range in South and Southeast Asia (India, Sri Lanka, Bangladesh, Indonesia, China, Japan and Russia) with a tMRCA of 5600 (95% HPD: 4300–6900) years ago. The East Asian group C1a1-5 comprised haplotypes from southern China and Japan and one from the Russian Far East, with a tMRCA of 3500 (95% HPD: 2500–4700) years ago.

MJ networks were constructed using the mitogenome sequences of MUS-1 and CAS-1 (Supplementary Fig. S3). Cluster M1a7-3J showed an apparent star-like pattern, suggesting rapid population expansion. Clusters C1a and C1a1-5 exhibited a star-like shape. Other clustering patterns did not exhibit an apparent star-like structure. We performed mismatch distribution analysis using the sequence datasets, M1a7-3, C1a1 and C1a1–5, and the results showed signs of rapid expansion in the previous datasets of *Cytb* sequences (Suzuki et al. 2013; Kuwayama et al. 2017); we also used the M1a, C1a and M1a7-3J datasets, which showed signs of rapid expansion, as evidenced by unimodal curves of pairwise differences and nonsignificant raggedness indexes (Supplementary Fig. S4 and Table 2). Tajima’s *D* and/or Fu’s *F*s were significantly negative in these clusters, including M1a7-3J and C1a1, except C1a1-1, suggesting past population expansion (Table 2). We calculated the starting times of the expansion events using the obtained *τ* values and possible evolutionary rates of 0.9 × 10^−7^, 1.1 × 10^−^^7^, 2.0 × 10^−7^ and 4.0 × 10^−7^ substitutions/site/year (Table 2). The values obtained for M1a7-3J and C1a1 using the evolutionary rate of 1.1 × 10^−7^ substitutions/site/year were 2700 and 4000 years ago, respectively.

**Table 2:** Estimates of expansion start times (years before present) for each haplotype group based on mitochondrial genome sequences (16,038 bp).

Haplogroup	Major geographic range	*n*	Pi (%)	Tajima’s *D*	Fu’s *F*s	*τ* (confidence interval)	SSD	*r*	Expansion start time (years ago)^a^			
									0.9	1.1	2.0	4.0
MUS-1a	Northern Eurasia	41	0.023	−2.066**	−11.09**	44.056 (33.035–50.957)	0.00272	0.00191	15,293	12,486	6867	3433
M1a7-3	Korea and Japan	18	0.073	−2.336**	−9.189**	9.255 (7.572–12.994)	0.0076	0.01452	3213	2615	1442	719
M1a7-3J	Japan	13	0.056	−2.287**	−6.196**	9.523 (6.727–11.133)	0.03134	0.03386	3306	2699	1484	742
C1a	India, SE Asia, E Asia	30	0.11	−1.977**	−7.082*	19.796 (14.488–22.164)	0.00606	0.00534	6825	5584	2127	1535
C1a1	India, SE Asia, E Asia	24	0.092	−1.565*	−1.115	14.219 (10.875–16.121)	0.01369	0.00726	4936	4030	2216	1108
C1a1-5	Japan and Yunnan	11	0.056	−0.965	−0.531	11.586 (6.453–13.559)	0.03358	0.05058	4021	3284	1806	903

The significance of rapid expansion was assessed using the neutrality tests. Values of *τ*, the sum of the squared deviations (SSD), and raggedness (*r*) were calculated with Arlequin 3.5.1 software (Excoffier and Lischer 2010). *P* values of <0.05 and <0.01 are marked with * and **, respectively.

*P < 0.05; **P < 0.01

^a^Four possible evolutionary rates (×10^−7^ substitutions/site/year) were used to determine the expansion times; 0.9 (this study), 1.1 (Suzuki et al. 2015), and two addinal rates of 2.0 and 4.0 (e.g., Rajabi-Maham et al. 2008).

## Discussion

### Divergences between and within subspecies-specific mtDNA lineages

The present dataset of 98 complete mitogenome sequences (ca.16,000 bp) provides new insights into the evolution of *M*. *musculus*, revealing an overall picture of mtDNA lineage differentiation and tracking the prehistoric movements at higher resolution than any previous attempts. The NN network revealed simultaneous divergences in the four subspecies lineages, MUS, CAS, DOM and NEP (Supplementary Fig. S2), with estimated divergence times of 520,000–570,000 derived from the BEAST analysis. Simultaneous divergence was observed within the CAS lineage, with tMRCAs estimated ca. 230,000–260,000 years ago (Fig. 2). In addition, the lineage differentiations within MUS and NEP were estimated to occur 148,000 and 146,000 years ago, respectively. For the two distinct sublineages within CAS-3, the tMRCA was 164,000 years ago (Fig. 2). These events may be linked to consequences of geological events such as the penultimate glacial maximum (PGM) and subsequent rapid warming (135,000 years ago), as suggested for wood mice in temperate forests (Suzuki et al. 2015). Thus, the late Pleistocene global environmental fluctuation of the 100,000-year cycle was likely involved in the simultaneous divergence events of *M*. *musculus* within their home range. Investigation of this hypothesis would provide useful clues to the evolutionary processes of grassland species in a subtropical region without the influence of human activities.

The mitogenome sequences show that the numbers of nucleotide substitutions among the five DOM haplotypes from Russia are comparable with those associated with the divergence of MUS-1 and MUS-2 146,000 years ago, as described above. The current genetic diversity of mtDNA in DOM (genetic distance: 0.0056–0.067 among the four distinct haplotypes) has presumably resulted from rapid expansion event that occurred in 130,000 years ago after the PGM. Meanwhile, inhabitation of western Europe by *M*. *musculus* is thought to have occurred ca. 15,000 years ago (Rajabi-Maham et al. 2008; Förster et al. 2009; Auffray and Britton-Davidian 2012; García-Rodríguez et al. 2018). Whereas the time estimation with the rapid evolutionary rate (e.g., 2.0 × 10^−^^7^ substitutions/site/year) indicates a more recent expansion event with a predicted initiation time of 15,000 years ago (Rajabi-Maham et al. 2008), in our view the range expansion of DOM may have involved mice with a substantial amount of genetic diversity that did not experience an efficient bottleneck event, instead involving colonisation from the source area to western Europe.

### Stepwise eastward movement of MUS mtDNA

**Fig. 6:**
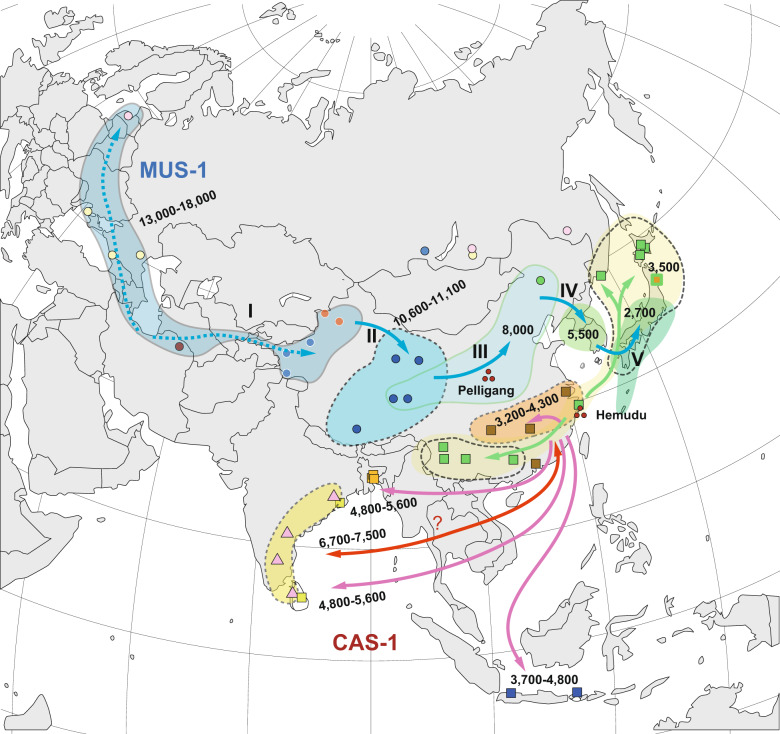
Schematic representation of the early prehistoric eastward movements of two subspecies of *Mus musculus*, MUS and CAS, as predicted from the mitogenome sequences. The movement of MUS was achieved mainly by the sublineage MUS-1 and involved five historical processes (Steps I–V): (1) the initial spread of MUS-1 to northern Eurasia, including westernmost western China, (2) movement to the eastern part of western China, (3) expansion to northern China, (4) introduction to the Korean Peninsula and (5) colonisation of Japan by a descendant lineage of the Korean haplotype group. Eastward movement of the CAS lineage was conducted by the sublineage CAS-1, resulting in geographic coverage to the northernmost part of China by 9000 years ago. The second CAS-1 dispersal event is movement either from the eastern coast of India to southern China or vice versa. The next step is a simultaneous dispersal event from southern China to several peripheral regions, including the eastern coast of India, Sri Lanka and Bangladesh. The final step is dispersal from southern China to the Japanese Archipelago, Russian Far East and Yunnan, China. Possible times (×1000 years ago) of the dispersal events are shown.

The present study provides information on the evolutionary scenarios driving dispersal events associated with prehistoric human movements, in particular those related to the eastward movement of MUS and CAS (see Fig. 6 for summary). The use of whole mtDNA sequences (ca. 16,000 bp) allowed us to assess the phylogenetic relationships among haplotypes at a relatively high resolution. Using the 1.1 × 10^−7^ evolutionary rate, we can propose an evolutionary history that agrees with archaeological events with known dates, as discussed below. On the other hand, the use of the highly accelerated evolutionary rate (e.g., 4.0 × 10^−^^7^; e.g., Rajabi-Maham et al. 2008) yielded results that are inconsistent with previous archaeological findings. In particular, it suggests that colonisation of the Japanese Archipelago occurred very recently (ca. 740 years ago, Table 2), which is not realistic. The divergence patterns of MUS-1 indicate that the basal offshoot is represented by the haplotype (sample code: MG3045) collected from central Sakhalin (termed M1b, Fig. 3). The genetic distance between M1a and M1b is ca. 0.005, and it is more plausible to use the intermediate evolutionary rate of 4.7 × 10^−8^, rather than 1.1 × 10^−7^, substitutions/site/year (Hanazaki et al. 2017). The divergence time is estimated to be ca. 54,000 years ago, corresponding to the early MIS 3, a critical period during the late Quaternary, in which population expansion events were triggered in wood mice and voles (Hanazaki et al. 2017; Honda et al. 2019). This may imply that the migration of MUS-1 mice (M1b) to Sakhalin via the land bridge occurred long ago, and that the mice have survived there up to present day. However, because the estimated divergence time is prior to the period of the last glacial maximum, it is hard to explain the ancient habitation of *M. musculus* in Sakhalin. This is more likely the consequence of secondary introduction during the modern age (i.e., during the last 100 years), as other haplotypes from Siberia (sample code: MG3004) and the Russian Far East (MG3026) contain almost identical sequences to those recovered from the westernmost sites in eastern Europe (MG3008).

The pattern of lineage divergence among the remaining haplotypes (M1a) is characterised by five episodic events (Steps I–V) of intermittent dispersal or population growth (Fig. 4): (1) colonisation of broad areas of eastern Europe, Central Asia and western China 13,400–18,300 years ago, (2) dispersal from the western to eastern parts of western China ca. 10,600–11,100 years ago, (3) emergence of diversity in northern China ca. 8000 years ago, (4) population growth in the Korean Peninsula ca. 4300–5500 years ago and (5) colonisation and sudden population growth in the Japanese Archipelago ca. 2700 years ago (Table 2). The initial range expansion of M1a broadly covered central and northern Eurasia, including western China (such as the Tarim Basin) (Fig. 4). M1a7, the easternmost sublineage among the seven haplogroups of M1a, includes three descendant lineages (M1a7-1–3) that show simultaneous divergence, suggesting population growth in northern China. The next episodic event is the emergence of the Korean lineage, termed M1a7-3. Subsequently, a sudden expansion event occurred in the Korean Peninsula. Finally, a single offshoot of M1a7-3 moved to Japan, resulting in a rapid population expansion (M1a7-3J).

The mitogenome sequence of the mouse (MG3007) from Ukraine was found to cluster with those from the eastern part of the western China (M1a7-1, Step II in Fig. 4). This can be explained either by ancient (e.g., 7500 year ago) or recent (e.g., the modern age) translocation from western China to Ukraine. The latter may be more likely, since the modern age transportation has been denoted in *M. musculus*. For example, recent stowaway introductions of DOM mice (e.g. 50 generations ago) to the Japanese Archipelago are evident in haplotype structure analyses on the nuclear genome (Nunome et al. 2010; Kuwayama et al. 2017).

While eastward population movements have been shown in previous studies (Nunome et al. 2013; Suzuki et al. 2013; Jing et al. 2014; Kuwayama et al. 2017), our mitogenome data provide clearer branching patterns and more reliable divergence time estimates, allowing construction of a hypothetical framework for the prehistoric movements of MUS in Eurasia, which exhibits good accordance with archaeological reports (Fig. 4b). The first two steps (Steps I and II) are attributable to the end of the last Ice Age (ca. 15,000 years ago) and the end of the Younger Dryas (11,600 years ago), but it remains unclear whether these are directly related to environmental changes or were consequences of Neolithic human geographic expansion.

The remaining three steps (Steps III–V) involved sudden population expansions in northern China, Korea and Japan, and agriculture-driven environmental change is considered a major factor shaping these sudden regional population expansions (e.g., Nelson et al. 2020). Mouse colonisation 7000–9000 years ago in the eastern region of northern China is supported by archaeological evidence from the unique culture called Peiligang Culture, which is characterised by the cultivation of millet (Zheng et al. 2009, 2012). Millet farming is considered to have transferred to the Korean Peninsula (Middle Chulman) no later than 5,400 years ago (Crawford and Lee 2003; Lee et al. 2011; Miyamoto 2016). Robust archaeological evidence supports the introduction of irrigated rice cultivation to Japan in the early Yayoi period ca. 3000 years ago (e.g., Crawford and Lee 2003; Fuller 2011; Miyamoto 2016). Notably, at the same time that *M. musculus* diversified in the Japanese Archipelago, an expansion-like event occurred in the Korean Peninsula, as suggested by the ML tree (Stage V, Fig. 4) and MJ network (Supplementary Fig. S3A). This suggests that historical social and agricultural developments took place on the Korean peninsula (early to middle Mumun pottery period) and Japanese Archipelago (early Yayoi period) simultaneously.

### Multiple possible expansions of CAS in southern China

Phylogeographic analysis showed that the divergence patterns of CAS-1 consist of two ancient divergences: one for the split in northernmost China (C1b) and the other for the split in the Indian (Sri Lanka) haplogroup (C1a2) with tMRCAs of ca. 8800 and 7500 years ago, respectively (Fig. 6). The divergence patterns suggest that CAS-1 mice became widespread in the broad area of Asia, extending to the northernmost part of China and the central and southern parts of the Indian subcontinent up to 9000 years ago. This range extension can be explained by two migration events from India to China. Alternatively, this pattern can be explained by multiple range expansion events from a central region, namely southern China. Mismatch distribution analysis revealed a sign of the rapid expansion in C1a1, which was estimated to have started ca. 4000 years ago, based on the evolutionary rate of 1.1 × 10^−^^7^ substitutions/site/year (Table 2). The *M. musculus* radiation event implies multiple historical human movements across the sea in the broad area of southern China and its surrounding areas, including India, Sri Lanka, Bangladesh and Indonesia (Fig. 6).

The expansion events predicted in Southeast Asia and East Asia coincide with the early stages of agricultural development in Asia (Diamond and Bellwood 2003; He et al. 2017). From the historical context of the Hemudu archaeological site in China, where rice cultivation is thought to have begun 7000 years ago, it is possible to assume that the initial expansion event of CAS mice is related to rice cultivation. In this study, we obtained two signs of rapid expansion events for C1a and C1a1 in the mitogenome data of CAS-1 recovered from southern Asia, with estimated initiation times of 5600 and 4000 years ago, respectively (Table 2). The haplotypes from Java and Bali showed a divergence time of 3700 years ago (Fig. 5), indicative of ancient colonisation of remote islands. This event may be linked with colonisation by Austronesian people, who began to spread across the Indonesian Islands 4000 years ago (Bulbeck 2008).

### Implications of mouse migrations for the ‘inner-dual structure model’

When Yonekawa et al. (1988) reported the coexistence of *M. m. musculus* type and *M. m. castaneus* type mtDNA in Japanese mice, they suggested a plausible connection to the model of human evolution in the Japanese Archipelago. Later, this human evolution model was called the ‘dual structure model’ by Hanihara (1991). The major players in this model are indigenous Jomon hunter–gatherers and later Yayoi migrants, who migrated around 2900 years ago (Habu 2004) and cultivated rice. *M. m. castaneus* was assumed to arrive to the Japanese Archipelago with the Jomon people, and *M. m. musculus* was assumed to arrive to the Japanese Archipelago with Yayoi migrants. Kanzawa-Kiriyama et al. (2019) estimated, using the newly determined Jomon genome, that the Ainu people in Hokkaido and the Okinawa people in the Ryukyu islands transmitted much higher proportions (~60% and ~25%, respectively) of the Jomon genome, compared with the mainland Japanese (~10%). This pattern is consistent with Hanihara’s (1991) ‘dual structure model’.

In this study, based on mouse mitogenome data, we estimated the migration dates of mouse population movements of CAS-1 and MUS-1 to the Japanese Archipelago to be ca. 3500 and 2700 years ago, respectively (see Fig. 6). The migration of *M. m. castaneus* type mtDNA was relatively recent, yet during the late Jomon period. These two mouse migration periods are consistent with Saitou’s (2017) second and third migration periods to the Japanese Archipelago, according to his three-layer migration model. It should be noted that the first migration period in this model spans from 40,000 to 4500 years ago. Based on these two layers of human migration, Saitou and Jinam (2017) proposed the ‘inner-dual structure model’ of the mainland Japanese; descendants of second-layer migrants spread all over mainland Japan, whereas descendants of third-layer migrants are distributed mainly in the central axis of the mainland (see Saitou 2017). Whether this model is consistent with the human and mouse DNA diversity patterns on mainland Japan is unknown.

## Conclusion

The early and middle Holocene is a crucial time period, when the fundamental regional human populations and cultures were established through colonisation and transfer of agricultural systems. In this context, the house mouse *M*. *musculus* can be expected to provide valuable information on human colonisation and agricultural development, as shown in previous studies (e.g., Nunome et al. 2010; Jones et al. 2013; Kodama et al. 2015). This study provided several new insights into the spatiotemporal dynamics of *M*. *musculus* based on whole mitogenome sequences. In particular, the divergence time estimates from our study are consistent with those reported in previous archaeological studies (Crawford and Lee 2003; Fuller et al. 2010, 2014; Fuller 2011; Miyamoto 2016). Here, we traced the stepwise colonisation of MUS-1 mice from portions of northern Eurasia, including western China, eastward through northern China to Korea, and finally to Japan. On the other hand, CAS-1 is important because it provides clear evidence of human exchange between the continent and the Japanese Archipelago. The new insights from this study are expected to be useful for improving interpretation of archaeological evidence. This accordance indicates that use of the entire mitogenome sequence and an evolutionary rate of 1.1 × 10^−^^7^ substitutions/site/year, as in this study, can provide relatively reliable estimates of divergence patterns and times for rodents in the Holocene.

## Supplementary information

Supplementary figures

Supplementary Figure Table S1

## Data Availability

Newly obtained DNA sequences were deposited in the DDBJ/EMBL/GenBank database with accession numbers LC552831–LC552928. DNA alignments have been deposited in Dryad: 10.5061/dryad.zkh189384.
