# Supplementary material for: House mouse Mus musculus dispersal in East Eurasia inferred from 98 newly determined complete mitochondrial genome sequences
Source: Heredity (Edinb). 2020 Sep 15;126(1):132–47. doi: 10.1038/s41437-020-00364-y (PMC7852662; doi:10.1038/s41437-020-00364-y)

Supplementary Fig. S1. (**A**) Scatter plot showing the relationship between the genetic distances (*p*-distance) of the mitochondrial cytochrome *b* (*Cytb*, 1140 bp) and whole genome sequences (16,038 bp) based on 98 mouse mitogenome sequences, indicating that the former is ~80% of the latter. Given that the evolutionary rate of *Cytb* is 3.0×10^{-8} substitutions/site/year (e.g. Honda et al. 2019), that of the whole mitogenome sequence is estimated be 2.4×10^{-8} substitutions/site/year. The *p*-distances of Nodes *a*, *b* and *c* (see Supplementary Fig. S2) are marked. (**B**) For comparison, points representing the genetic distances (*p*-distance) of *Cytb* and predicted divergence times in the large Japanese wood mouse (blue diamonds, *Apodemus speciosus*; Hanazaki et al. 2017) and *Mus musculus* (red circle, overall mean distances for each of Nodes *a*, *b* and *c*) are shown.

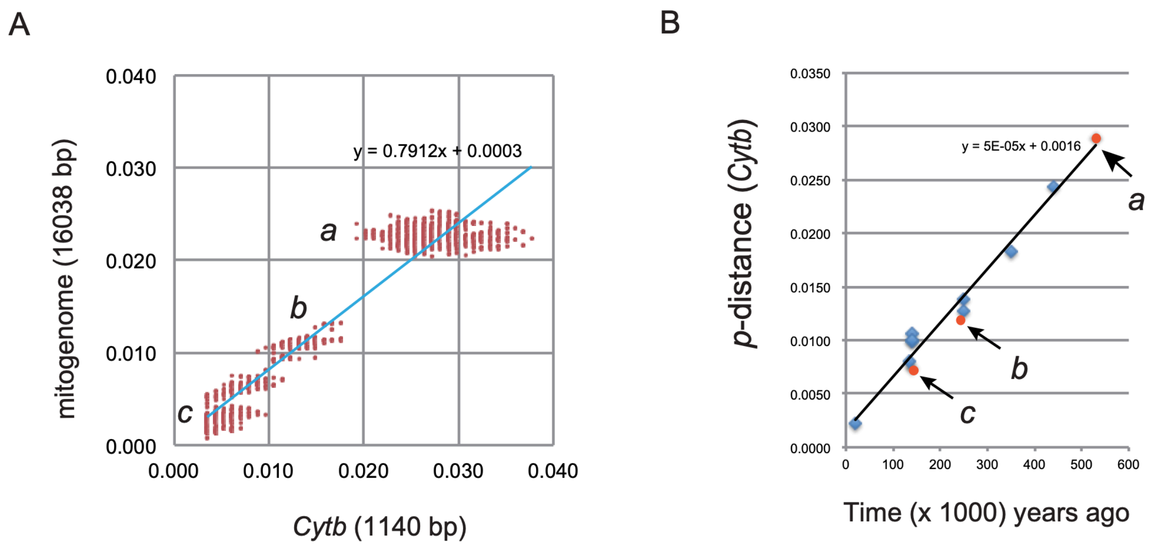

Supplementary Fig. S2. Neighbor-net network tree based on the whole mitochondrial sequences (16,038 bp), with tip labels for the three major subspecies groups, *Mus musculus musculus* (MUS), *M. m. castaneus* (CAS) and *M. m. domesticus* (DOM) and a geographically confined group of Nepalese mice (NEP). Distinct sublineages are labelled. The aggregates of multiple nodes and node are marked (*a*, *b*, *c*).

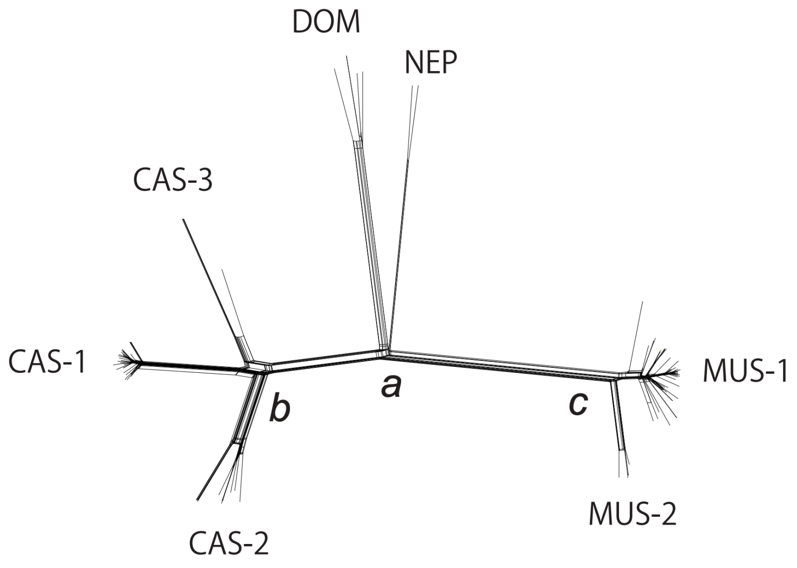

Supplementary Fig. S3. (A) Median-joining haplotype network based on the whole mitochondrial DNA sequences of the northern Eurasian subspecies, *Mus musculus musculus*. The number of mutations between haplotypes is indicated with dots and numbers. Circle size is related to haplotype frequency. The groups within the shaded area correspond to the haplogroups listed in Fig. 4A. **(B)** Median-joining haplotype network based on the whole mitochondrial DNA sequences of the southern Eurasian subspecies, *Mus musculus castaneus*. The groups within the shaded area correspond to the haplogroups shown in Fig. 5A.

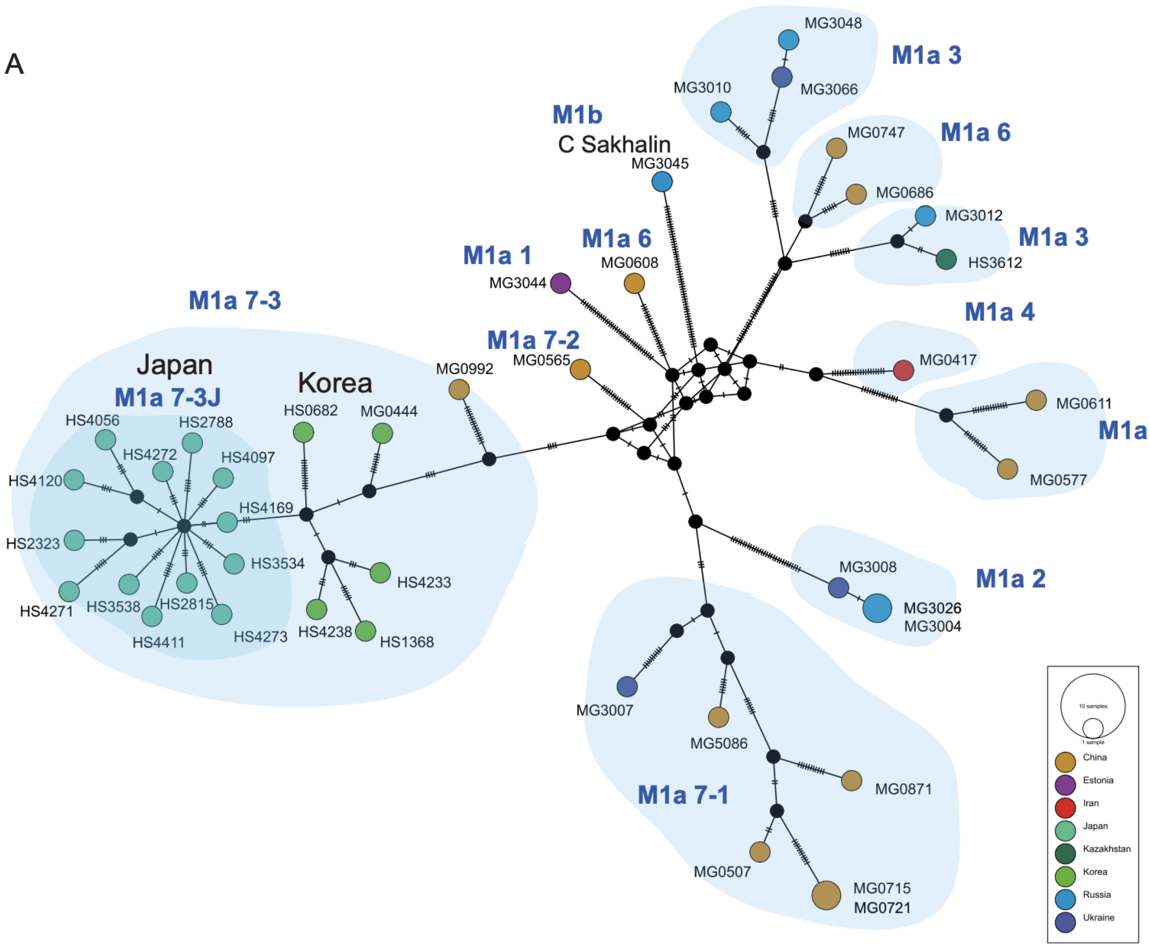

B

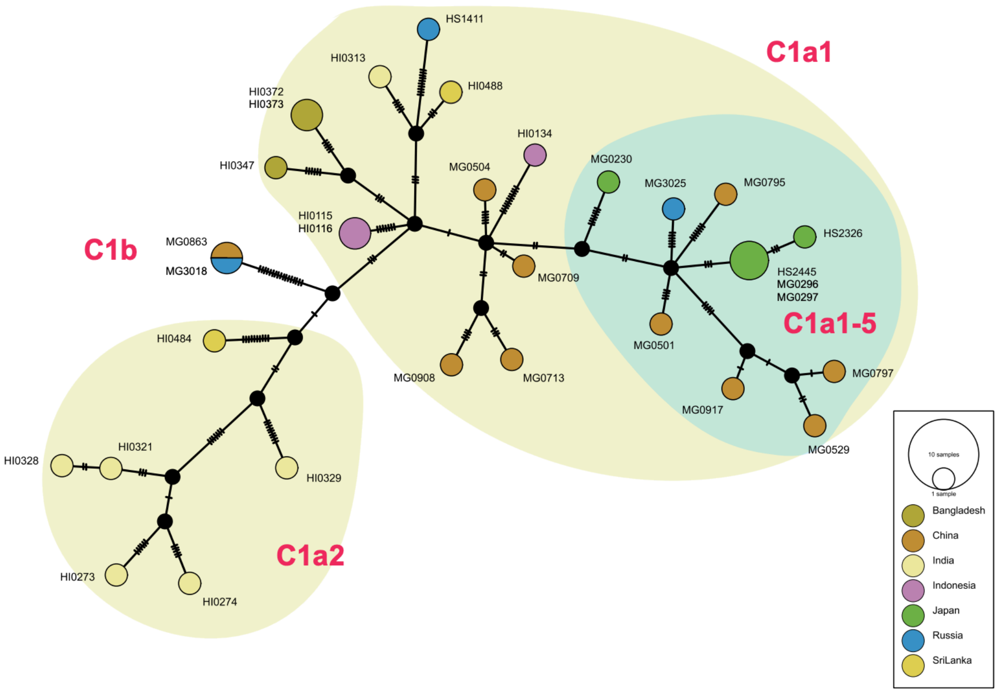

C1a1
C1a1-5
C1b
C1a2
HS1411
HI0313
HI0488
HI0372
HI0373
HI0347
HI0134
MG0504
MG0230
MG3025
MG0795
HS2326
HI0115
HI0116
MG0863
MG3018
MG0709
HS2445
MG0296
MG0297
MG0501
HI0484
MG0908
MG0713
MG0797
MG0917
MG0529
HI0321
HI0328
HI0329
HI0273
HI0274
10 samples
1 sample
Bangladesh
China
India
Indonesia
Japan
Russia
SriLanka

Supplementary Fig. S4. Mismatch distribution of the whole mitochondrial sequences (16,038 bp). Only clades or subclades that showed significant evidence of expansion are shown. Bars indicate the observed frequencies of mutations between haplotypes and the line denotes the expected frequency under the sudden expansion model.

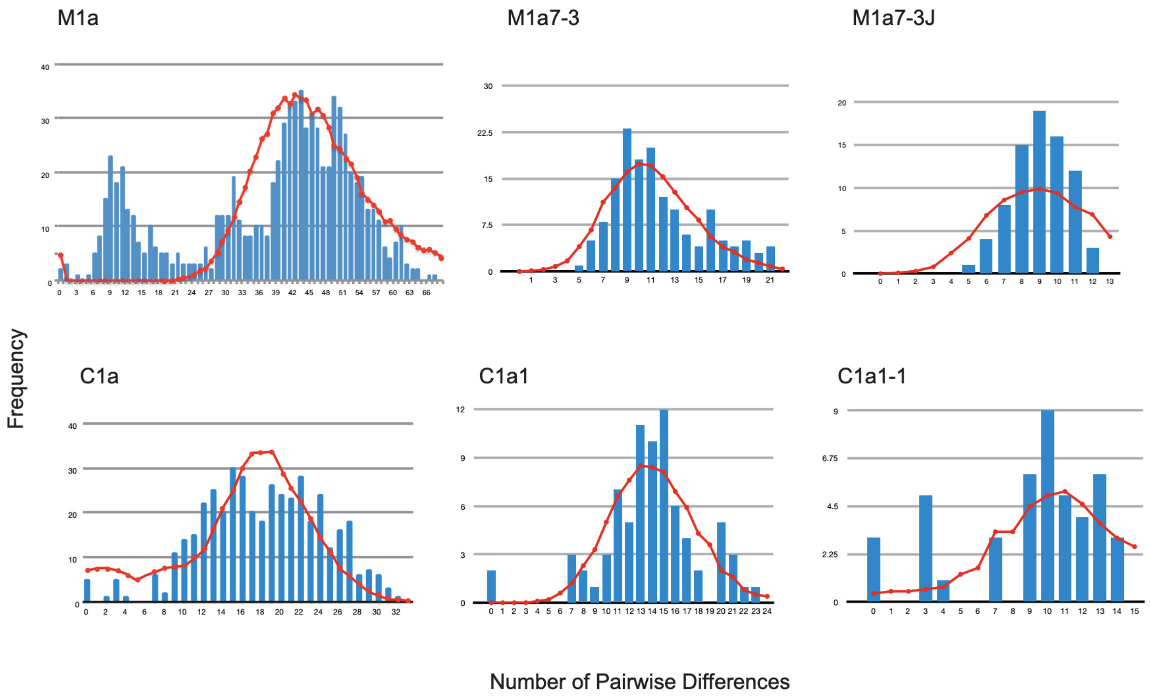

Supplement: Supplementary file 1 — Supplementary figures [file 41437_2020_364_MOESM1_ESM.pdf]
