# Supplementary material for: House mouse Mus musculus dispersal in East Eurasia inferred from 98 newly determined complete mitochondrial genome sequences
Source: Heredity (Edinb). 2020 Sep 15;126(1):132–47. doi: 10.1038/s41437-020-00364-y (PMC7852662; doi:10.1038/s41437-020-00364-y)
Supplement: Supplementary file 2 — Supplementary Figure Table S1 [file 41437_2020_364_MOESM2_ESM.pdf]

Supplementary Table S1. List of samples used in this study and their mitochondrial genotyping.

No.	DNA No.	Country&Area	Subspecies phylogroup	Subgroup	Sex
1	MG3044	Estonia:Tallinn	MUS	1	M
2	MG3007	Ukraine:Kremenets	MUS	1	M
3	MG3008	Ukraine:Kremenets	MUS	1	F
4	MG3066	Ukraine:Donetsk	MUS	1	M
5	MG3056	Russia：Moscow	DOM		F
6	MG3010	Russia:North Caucasus	MUS	1	M
7	HS3604	Russia:Tomsk	DOM		F
8	HS3605	Russia:Gorno-Altaysk	MUS	2	M
9	HS3608	Russia:Irkutsk	MUS	2	M
10	MG3004	Russia:Chita	MUS	1	F
11	MG3012	Russia:Chita	MUS	1	F
12	MG3026	Russia:Birakan	MUS	1	M
13	MG3037	Russia：Khabarovsk	DOM		F
14	HS1411	Russia:Khasan	CAS	1	unknown
15	MG3025	Russia:Vladivostok	CAS	1	F
16	MG3018	Russia:Rudnaya	CAS	1	M
17	HS3607	Russia:Okha	DOM		M
18	MG3045	Russia:Poronaysk	MUS	1	F
19	MG3048	Russia:Yuzhno-Sakhalinsk	MUS	1	F
20	HS1464	Kazakhstan:Aktobe	MUS	2	M
21	HS3612	Russia:Astrakhan	MUS	1	M
22	MG5135	Iran:Now Shahr	CAS	2	F
23	MG0417	Iran:Mashhad	MUS	1	M
24	HI173	Pakistan:Islamabad	CAS	2	F
25	HI175	Pakistan:Islamabad	CAS	3	F
26	HI264	Pakistan:Lahore	CAS	2	M
27	HI261	Pakistan:Sahiwal	CAS	2	M
28	MG0686	China:Kashi	MUS	1	M
29	MG0747	China:Hotan	MUS	1	F
30	MG0608	China:Aksu	MUS	1	M
31	MG611	China:Tacheng	MUS	1	M
32	MG597	China:Manasi	MUS	2	M
33	MG577	China:Urumqi	MUS	1	F
34	MG715	China:Lasa	MUS	1	M
35	MG721	China:Lasa	MUS	1	F
36	MG0871	China:Dunhuang	MUS	1	M
37	MG5086	China:Jiayuguan	MUS	1	M
38	MG565	China:Xining	MUS	1	F
39	MG917	China:Lijiang	CAS	1	M
40	MG797	China:Dali	CAS	1	M
41	MG507	China:Lanzhou	MUS	1	M
42	MG529	China:Kunming	CAS	1	M
43	MG709	China:Chongqing	CAS	1	F
44	MG0501	China:Guilin	CAS	1	F
45	MG0908	China:Wuhan	CAS	1	M
46	MG504	China:Guangzhou	CAS	1	M
47	MG863	China:Manzhouli	CAS	1	M
48	MG0631	China:Mohe	MUS	2	F
49	MG713	China:Chinkiang	CAS	1	M

To be continued

Table S1 (continued)

50	MG992	China:Qiqihar	MUS	1	F
51	MG795	China:Ningbo	CAS	1	F
52	HI159	India:Leh	CAS	3	M
53	HI161	India:Leh	CAS	3	M
54	HI185	India:Delhi	CAS	2	F
55	HI187	India:Delhi	CAS	2	F
56	HI328	India:Hyderabad	CAS	1	unknown
57	HI329	India:Hyderabad	CAS	1	unknown
58	HI273	India:Mysore	CAS	1	F
59	HI274	India:Mysore	CAS	1	F
60	HI321	India:Bhubaneswar	CAS	1	M
61	HI313	Inida:Bhubaneswar	CAS	1	F
62	HS1467	Nepal:Tukuche	NEP	-	M
63	HS1523	Nepal: Kathmandu	NEP	-	M
64	HI488	Sri Lanka:Colombo	CAS	1	M
65	HI484	Sri Lanka:Peradeniya	CAS	1	M
66	HI347	Bangladesh:Mymensingh	CAS	1	F
67	HI372	Bangladesh : Dhaka	CAS	1	F
68	HI373	Bangladesh : Dhaka	CAS	1	F
69	HI505	Vietnam:Vinh Phu	CAS	2	M
70	HI520	Vietnam:Hanoi	CAS	2	M
71	HI134	Indonesia:Lembang	CAS	1	M
72	HI112	Indonesia:Bogor	DOM	-	M
73	HI115	Indonesia:Bali	CAS	1	F
74	HI116	Indonesia:Bali	CAS	1	F
75	HS2400	Taiwan:Taitung	CAS	2	M
76	HS682	Korea:Baengnyeong I.	MUS	1	M
77	HS4238	Korea:Ganghwa I.	MUS	1	F
78	HS4233	Korea:Hwacheon-gun	MUS	1	unknown
79	HS1368	Korea:Konchon	MUS	1	M
80	MG444	Korea:Busan	MUS	1	M
81	HS2326	Japan:Nayoro	CAS	1	M
82	HS2445	Japan:Takikawa	CAS	1	M
83	MG0296	Japan:Teine, Sapporo	CAS	1	M
84	MG0297	Japan:Teine, Sapporo	CAS	1	F
85	HS4271	Japan:Setana	MUS	1	unknown
86	HS2323	Japan:Hakodate	MUS	1	M
87	MG0230	Japan:Ashiro, Iwate	CAS	1	M
88	HS3538	Japan:Morioka, Iwate	MUS	1	unknown
89	HS3534	Japan:Yamagata	MUS	1	unknown
90	HS4169	Japan:Tsukuba	MUS	1	unknown
91	HS2815	Japan:Nonoichi	MUS	1	M
92	HS4411	Japan:Ohta	MUS	1	unknown
93	HS4120	Japan:Ishii	MUS	1	F
94	HS4056	Japan:Fujisawa	MUS	1	F
95	HS4097	Japan:Misasa	MUS	1	M
96	HS4273	Japan:Yamaguchi	MUS	1	F
97	HS4272	Japan:Fukuoka	MUS	1	M
98	HS2788	Japan:Okinawa	MUS	1	unknown

*See Text and Fig. 2 for detail.
